# A two‐phase epigenome‐wide four‐way gene–smoking interaction study of overall survival for early‐stage non‐small cell lung cancer

**DOI:** 10.1002/1878-0261.13766

**Published:** 2024-12-04

**Authors:** Leyi Chen, Xiang Wang, Ning Xie, Zhongwen Zhang, Xiaowen Xu, Maojie Xue, Yuqing Yang, Liya Liu, Li Su, Maria Bjaanæs, Anna Karlsson, Maria Planck, Johan Staaf, Åslaug Helland, Manel Esteller, David C. Christiani, Feng Chen, Ruyang Zhang

**Affiliations:** ^1^ Department of Biostatistics, Center for Global Health, School of Public Health Nanjing Medical University China; ^2^ Department of Health Inspection and Quarantine, Center for Global Health, School of Public Health Nanjing Medical University China; ^3^ School of Public Health, Health Science Center Ningbo University China; ^4^ Department of Environmental Health Harvard T.H. Chan School of Public Health Boston MA USA; ^5^ Pulmonary and Critical Care Division, Department of Medicine Massachusetts General Hospital and Harvard Medical School Boston MA USA; ^6^ Department of Cancer Genetics, Institute for Cancer Research Oslo University Hospital Norway; ^7^ Division of Oncology, Department of Clinical Sciences Lund and CREATE Health Strategic Center for Translational Cancer Research Lund University Sweden; ^8^ Institute of Clinical Medicine University of Oslo Norway; ^9^ Josep Carreras Leukaemia Research Institute Barcelona Spain; ^10^ Centro de Investigacion Biomedica en Red Cancer Madrid Spain; ^11^ Institucio Catalana de Recerca i Estudis Avançats Barcelona Spain; ^12^ Physiological Sciences Department, School of Medicine and Health Sciences University of Barcelona Spain; ^13^ China International Cooperation Center for Environment and Human Health Nanjing Medical University China; ^14^ Changzhou Medical Center Nanjing Medical University Changzhou China; ^15^ Information Center The Affiliated Changzhou Second People's Hospital of Nanjing Medical University Changzhou China

**Keywords:** epigenomics, four‐way interaction, gene–smoking interaction, NSCLC

## Abstract

High‐order interactions associated with non‐small cell lung cancer (NSCLC) survival may elucidate underlying molecular mechanisms and identify potential therapeutic targets. Our previous work has identified a three‐way interaction among pack‐year of smoking (the number of packs of cigarettes smoked per day multiplied by the number of years the person has smoked) and two DNA methylation probes (cg05293407_*TRIM27*_ and cg00060500_*KIAA0226*_). However, whether a four‐way interaction exists remains unclear. Therefore, we adopted a two‐phase design to identify the four‐way gene–smoking interactions by a hill‐climbing strategy on the basis of the previously detected three‐way interaction. One CpG probe, cg16658473_*SHISA9*_, was identified with FDR‐*q* ≤ 0.05 in the discovery phase and *P* ≤ 0.05 in the validation phase. Meanwhile, the four‐way interaction improved the discrimination ability for the prognostic prediction model, as indicated by the area under the receiver operating characteristic curve (AUC) for both 3‐ and 5‐year survival. In summary, we identified a four‐way interaction associated with NSCLC survival among pack‐year of smoking, cg05293407_*TRIM27*_, cg00060500_*KIAA0226*_ and g16658473_*SHISA9*_, providing novel insights into the complex mechanisms underlying NSCLC progression.

## Graphical abstract

This study identifies a four‐way interaction among smoking pack‐years and three DNA methylation probes (cg05293407_*TRIM27*_, cg00060500_*KIAA0226*_, and cg16658473_*SHISA9*_) associated with NSCLC survival. This interaction improves the model's ability to predict survival outcomes, offering new insights into molecular mechanisms of NSCLC progression and potential therapeutic targets.

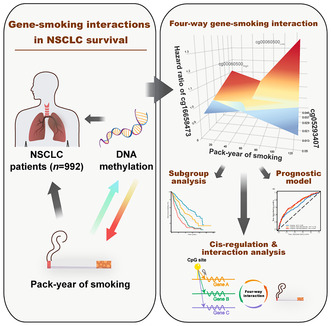

**Abbreviations**
AUC: area under the receiver operating characteristic curve
CI: confidence interval
*C*‐index: concordance index
FDR: false discovery rate
G × E interaction: gene–environment interaction
G × G interaction: gene–gene interaction
GEO: Gene Expression Omnibus
GO: gene oncology
HR: hazard ratio
IKKε: the inhibitor of nuclear factor κB kinase subunit epsilon
KEGG: Kyoto Encyclopedia of Genes and Genomes
LUAD: lung adenocarcinoma
LUSC: lung squamous cell carcinoma
NSCLC: non‐small cell lung cancer
QC: quality control
ROC: receiver operating characteristic
SD: standard deviation
SNP: single nucleotide polymorphisms
TCGA: The Cancer Genome Atlas
TFEB: transcription factor e‐box binding
TRIUNE: integrated covariates and four‐way interaction score

## 1 Introduction

Lung cancer is one of the most prevalent malignancies worldwide and a leading cause of cancer‐related mortality. According to global cancer statistics, almost 2.5 million new cases are diagnosed and 1.8 million new deaths are registered annually [1]. Most of lung cancer patients were non‐small‐cell lung cancer (NSCLC) [2, 3], of which two major histological subtypes were lung adenocarcinoma (LUAD) and lung squamous cell carcinoma (LUSC) [4, 5]. Nowadays, lung cancer survival rates remain low, with a 3‐year survival rate of 13–40% and a 5‐year rate around 25% [2]. However, various clinical outcomes are observed among early‐stage NSCLC patients, even among those with similar clinical characteristics [6]. This indicates that there are still hidden mechanisms behind the development of lung cancer.

Epigenetic alterations, notably DNA methylation, are recognized as key factors regulating molecular mechanisms of tumorigenesis and cancer progression [7, 8]. As a reversible epigenetic modification process, DNA methylation primarily involves the substitution of the 5′‐hydroxyl group of DNA with a methyl group, known as 5‐methylcytosine (5mC). This modification holds promise as a tool for early detection [9, 10], providing pivotal insights into tumor metastasis and prognosis [11, 12] and guiding therapeutic strategies, including immunotherapy and chemotherapy [6, 13, 14], for NSCLC patients. Additionally, gene–gene (G × G) interactions between methylation traits have been reported for their pivotal insights into the biological mechanisms underlying NSCLC [15, 16, 17]. Over the past two decades, epigenetic characteristics have been proved to be influenced by environmental exposure such as smoking, which affects the course of cancer progression [18]. This complex interaction pattern between smoking and DNA methylation, referred to as the gene–environment (G × E) interaction, parallels G × G interactions, both recognized for their crucial roles in elucidating the intricate molecular mechanisms underlying various complex diseases [19, 20], including NSCLC [21, 22, 23]. Our previous studies have found the patterns under two‐way interaction (pack‐year of smoking × cg05293407_*TRIM27*_) and three‐way interaction (pack‐year of smoking × cg05293407_*TRIM27*_ × cg0060500_*KIAA0226*_) between DNA methylation and smoking. These patterns may reveal the histologically heterogeneous effect of molecular mechanisms of cancer development in NSCLC [22, 23]. Considering that the progression of tumors constitute a complex biological process characterized by multifaceted, multistep, multigenic, and multifactorial mechanisms, low‐order interactions are limited in correctly interpreting complex composite patterns related to cancer progression [24]. Higher‐order interactions deserve to be tested to confirm our previous work and explore new interactions, if they exist [25, 26]. Therefore, we performed the first epigenome‐wide four‐way gene‐smoking interaction study of NSCLC survival and identified one novel epigenetic biomarker associated with lung cancer prognosis.

In this study, we integrated the epigenomic and demographic data of multiple cohorts and employed a two‐phase study to elucidate the robust four‐way interaction influencing the overall survival of NSCLC patients. First, build upon the previously established three‐way interaction involving cg05293407_*TRIM27*_, cg00060500_*KIAA0226*_, and smoking, we conducted a four‐way interaction analysis involving pack‐year of smoking, cg05293407_*TRIM27*_, cg00060500_*KIAA0226*_ and an additional CpG probe, using samples from four international study centers (USA‐Harvard, Spain, Norway, Sweden). Second, we validated these significant signals by data obtained from the Cancer Genome Atlas (TCGA) database.

## 2 Materials and methods

### 2.1 Study populations of DNA methylation data

The DNA methylation data from early‐stage (stage I and II) NSCLC patients were collected from five international consortium: USA‐Harvard, Spain, Norway, Sweden study and TCGA database [27, 28, 29, 30]. Cases from USA‐Harvard, Spain, Norway, and Sweden study were assigned to discovery phase, while cases from TCGA were assigned to validation phase. All these studies were approved by each institutional review board, and patients provided written informed consent. The study methodologies conformed to the standards set by the Declaration of Helsinki and was approved by the local ethics committee.

#### 2.1.1 USA‐Harvard

The Harvard Lung Cancer Study cohort was described previously [27]. Patients were recruited at Massachusetts General Hospital between 1992 and 2016, and all were newly diagnosed and histologically confirmed with primary NSCLC at the time of recruitment. Snap‐frozen tumor samples were taken from patients during complete resection of the therapeutic procedure. A total of 149 early‐stage patients were selected in this study with complete survival information. Tumor DNA was extracted from 5‐μm‐thick histopathologic sections. Each specimen was evaluated by a pathologist for amount (tumor cellularity > 70%) and quality of tumor cells. All specimens were histologically classified using the Word Health Organization (WHO) criteria. The study was approved by the Institutional Review Boards of the Massachusetts General Hospital (Partners Human Research Committee, Protocol #1999P004935/MGH).

#### 2.1.2 Spain

As previously described [28], tumors were surgically collected from 207 early‐stage NSCLC patients between 1991 and 2009. These samples were obtained from several research institutions, including Spain (Catalan Institute of Oncology; Center for Applied Medical Research; and Bellvitge Biomedical Research Institute), Italy (IRCCS Foundation National Cancer Institute; and University of Turin), UK (University of Liverpool Cancer Research Centre), France (CHU Albert Michallon), and the United States (University of Michigan Medical School). Patients provided written consent and tumors were surgically collected. This study was approved by the Bellvitge Biomedical Research Institute institutional review boards (PR055/10).

#### 2.1.3 Norway

There were 132 LUAD patients participated in Norway cohort with operable lung cancer tumors enrolled at Oslo University Hospital, Rikshospitalet, Norway, between 2006 and 2011 [29]. The project was developed with the approval of the Oslo University Institutional Review Board and regional ethics committee (S‐05307). All patients provided informed consent. Tumor tissues were snap‐frozen in liquid nitrogen and stored at −80 °C until DNA isolation.

#### 2.1.4 Sweden

Tumor DNA were collected from 36 patients with early‐stage NSCLC, at Skane University Hospital, Lund, Sweden [30]. DNA extraction was performed on tumor specimens (10 μm‐thick, tumor cellularity > 50%). The study was developed under the approval of the Regional Ethical Review Board in Lund, Sweden (Registration no. 2004/762 and 2008/702).

#### 2.1.5 TCGA

TCGA database consisted of 227 LUAD and 241 LUSC cases with complete DNA methylation, overall survival time and covariates data. Level‐1 HumanMethylation450 DNA methylation data (image data) of each patient were downloaded at October 1, 2015.

### 2.2 Quality control procedures for DNA methylation data

The quality control (QC) procedures outlined here have been consistently applied in our previous studies [16, 22, 23]. DNA methylation was profiled using the Infinium HumanMethylation450 BeadChip (Illumina Inc., San Diego, CA, USA). All studies adhered to the same QC procedures before the association analysis. Raw image data were converted into beta values (continuous numbers ranging from 0% to 100%) using the GenomeStudio Methylation Module V1.8 (Illumina Inc.) for background subtraction and control normalization. Unqualified probes meeting any one of the following criteria were excluded: (a) failed detection (*P* > 0.05) in more than 5% samples; (b) coefficient of variance (CV) < 5%; (c) methylated or unmethylated in all samples; (d) common single nucleotide polymorphisms (SNP) located in the probe sequence or 10‐bp flanking regions; (e) cross‐reactive probes or cross‐hybridizing probes [31]; or (f) did not pass quality control in all centers. Samples with > 5% undetectable probes or missing of pack‐year of smoking were excluded. Methylation signals were further processed for quantile normalization (*betaqn* function in R package *minfi*) as well as type I and II probes correction (*BMIQ* function in R package *lumi*), and were adjusted for batch effects (*ComBat* function in R package *sva*) according to the best pipeline by a comparative study [32]. The QC processes are detailed in Fig. S1.

### 2.3 Study populations and quality control of gene expression data

Gene expression data for early‐stage NSCLC were derived from the USA‐Harvard, Norway, Sweden, and TCGA database. While, Spanish study center did not profile gene expression data. The USA‐Harvard, Norway, Sweden and TCGA database had 26, 93, 34, 456 individuals, respectively. The TCGA workgroup completed the mRNA sequencing data processing and QC. Level 3 gene quantification data were downloaded from the TCGA data portal and were further checked for quality. Gene probes were excluded if the missing rate > 80%, and batch effects were corrected with *ComBat*. The expression value of each gene was transformed on log_2_ scale and standardized subsequently.

### 2.4 Study design and statistical analysis

#### 2.4.1 A two‐phase design for four‐way gene‐smoking interaction study

**Fig. 1:**
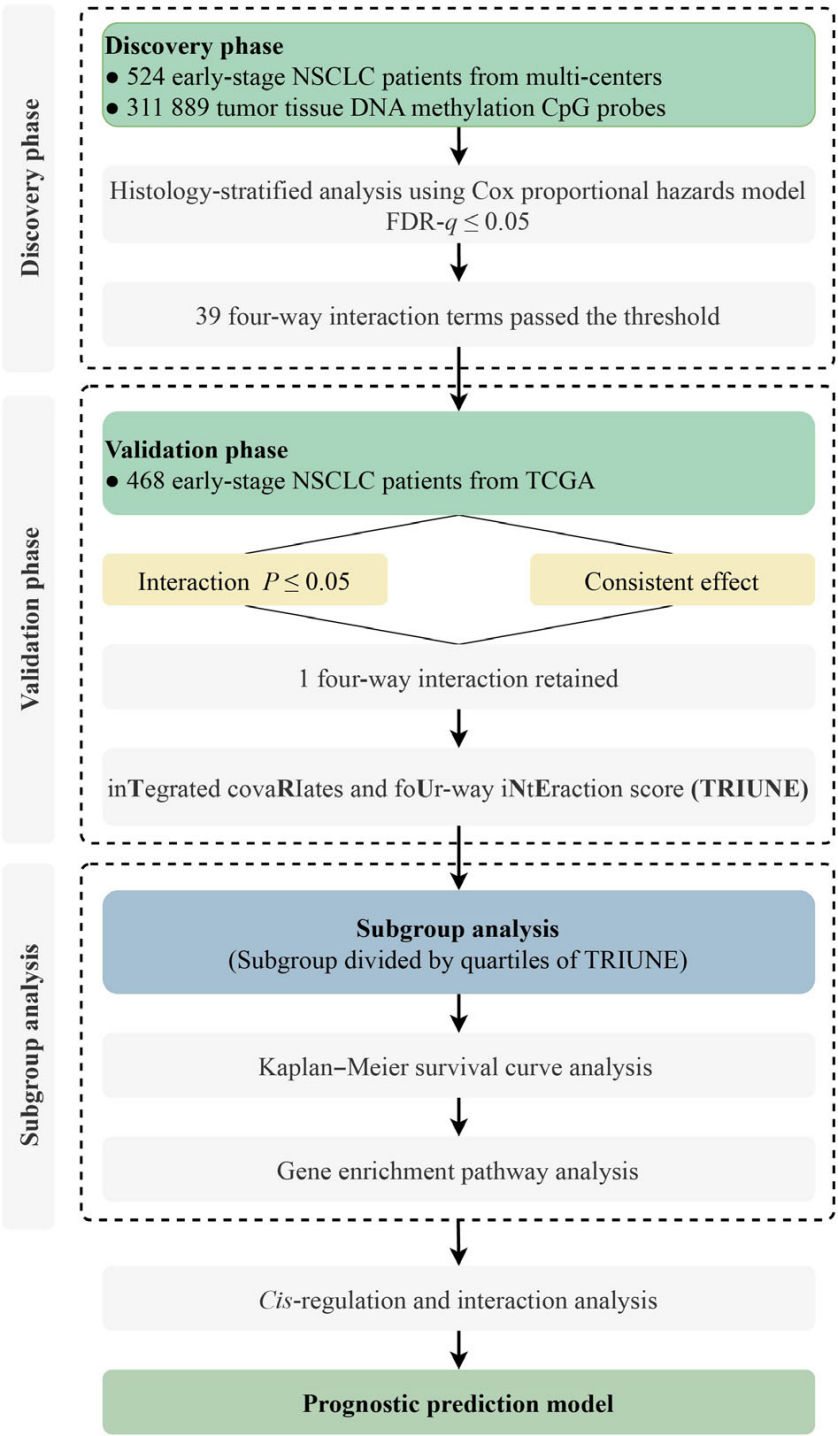
Flow chart of study design and statistical analysis. Patients diagnosed with lung adenocarcinoma (LUAD) and lung squamous cell carcinoma (LUSC) from the Harvard, Spain, Norway, and Sweden cohorts were utilized in the discovery phase for screening, whereas data from the Cancer Genome Atlas (TCGA) were employed for validation. Subgroup analyses were conducted based on quartiles of TRIUNE. FDR, false discovery rate; NSCLC, non‐small lung cancer.

The flowchart illustrating the analysis was depicted in Fig. 1, outlining a two‐phase design aimed at exploring the association between four‐way interactions and early‐stage NSCLC overall survival on an epigenome‐wide scale. In the discovery phase, we constructed a histology‐stratified Cox proportional hazards model adjusted for age, sex, smoking status, clinical stage, and study centers. In current study, we employed a hill‐climbing algorithm to detect high‐order interactions. Originally, we identified a significant two‐way interaction, i.e., pack‐year of smoking × cg05293407_*TRIM27*_, associated with NSCLC survival [22]. Then, we further recognized a significant three‐way interaction, i.e., pack‐year of smoking × cg05293407_*TRIM27*_ × cg0060500_*KIAA0226*_ [23]. Here, we aimed to identify another biomarker contributing to the significant four‐way interaction, i.e., pack‐year of smoking × cg05293407_*TRIM27*_ × cg0060500_*KIAA0226*_ × CpG probe, among samples from USA‐Harvard, Spain, Norway, and Sweden. This algorithm allows us to systematically explore significant signals from low‐order interaction to high‐order interaction [22, 23, 33]. Hazard ratio (HR) and 95% confidence interval (CI) was computed for per 1% level of the methylation increment. Multiple testing corrections were applied to control the false discovery rate (FDR) at a 5% level [34]. In the validation phase, the identified four‐way interactions will be tested in TCGA dataset. Significant interactions should meet the following criteria: (a) FDR‐*q* ≤ 0.05 in the discovery phase and *P* ≤ 0.05 in the validation phase; and (b) with consistent direction effect between two phases.

#### 2.4.2 TRIUNE subgroup analysis

An inTegrated covaRIates and foUr‐way iNtEraction score (TRIUNE) was defined by a weighted linear combination of all components inside the significant four‐way interaction, as well as all covariates, with weights derived from the aforementioned Cox proportional hazards model. Specifically,

$$\begin{array}{c} \text{TRIUNE}=\sum \alpha_{i}\times \text{Covariat}e_{i}+\beta_{1}\times \text{Pack}‐\text{year of smoking}+\beta_{2}\times \mathrm{cg}05293407_{\text{TRIM}27}+\beta_{3}\times \mathrm{cg}00060500_{\text{KIAA}0226}+\beta_{4}\times \mathrm{cg}16658473_{\text{SHISA}9}\\ \;+\beta_{12}\times \text{Pack}‐\text{year of smoking}\times \mathrm{cg}05293407_{\text{TRIM}27}+\beta_{13}\times \text{Pack}‐\text{year of smoking}\times \mathrm{cg}00060500_{\text{KIAA}0226}\\ \;+\beta_{14}\times \text{Pack}‐\text{year of smoking}\times \mathrm{cg}16658473_{\text{SHISA}9}\\ \;+\beta_{23}\times \mathrm{cg}05293407_{\text{TRIM}27}\times \mathrm{cg}00060500_{\text{KIAA}0226}+\beta_{24}\times \mathrm{cg}05293407_{\text{TRIM}27}\times \mathrm{cg}16658473_{\text{SHISA}9}\\ \;+\beta_{34}\times \mathrm{cg}00060500_{\text{KIAA}0226}\times \mathrm{cg}16658473_{\text{SHISA}9}\\ \;+\beta_{123}\times \text{Pack}‐\text{year of smoking}\times \mathrm{cg}05293407_{\text{TRIM}27}\times \mathrm{cg}00060500_{\text{KIAA}0226}\\ \;+\beta_{124}\times \text{Pack}‐\text{year of smoking}\times \mathrm{cg}05293407_{\text{TRIM}27}\times \mathrm{cg}16658473_{\text{SHISA}9}\\ \;+\beta_{234}\times \mathrm{cg}05293407_{\text{TRIM}27}\times \mathrm{cg}00060500_{\text{KIAA}0226}\times \mathrm{cg}16658473_{\text{SHISA}9}\\ \;+\beta_{1234}\times \text{Pack}‐\text{year of smoking}\times \mathrm{cg}05293407_{\text{TRIM}27}\times \mathrm{cg}00060500_{\text{KIAA}0226}\times \mathrm{cg}16658473_{\text{SHISA}9} \end{array}$$

where α_*i*_ and β_*i*_ were the coefficient estimated for each variable in the multivariable Cox proportional hazards model.

To further evaluate the prognostic value of the TRIUNE, we performed the following analyses. (a) Patients were divided into four subgroups by the quartiles of the TRIUNE, and their survival difference was compared using Kaplan–Meier survival curves. (b) To explore the molecular mechanisms underlying the TRIUNE, potential genes regulated by CpG probes involving in TRIUNE were identified by genome‐wide DNA methylation and gene expression correlation analysis using the linear regression model adjusted for the covariates aforementioned, with significant significance at FDR‐*q* ≤ 0.05. (c) Functional annotation and gene enrichment pathway analyses, including Gene Ontology (GO) and Kyoto Encyclopedia of Genes and Genomes (KEGG), were conducted to evaluate potential biological functions of significantly correlated genes using the *WebGestaltR* package [35, 36, 37, 38]. FDR‐*q* ≤ 0.05 was considered statistically significant.

#### 2.4.3 Four‐way gene‐smoking interaction analysis in gene expression level

We examined the four‐way gene‐smoking interaction in gene expression level using genes *cis*‐regulated by CpG probes. Specifically, the CpG probe located within 1 kb window of a gene and significantly (*P* ≤ 0.05) correlated with its expression were defined as candidate having significant epigenetic *cis*‐regulation of transcription. The four‐way gene‐smoking interaction at the gene expression level was tested by Cox model adjusted for the covariates aforementioned. Significant threshold was defined as *P* ≤ 0.05.

#### 2.4.4 Development of a prognostic prediction model

We utilized clinical variables and CpG probes involved in the four‐way interaction to developed a prognostic prediction model in the combined dataset. The discrimination ability of the prediction model was illustrated using time‐dependent receiver operating characteristic (ROC) curves, with the area under the ROC curve (AUC) calculated by *timeROC* package. The 3‐ and 5‐year overall survival time of cancer patients are well recognized as important clinical outcomes in practice [2, 39, 40]. Therefore, we gave 3‐ and 5‐year ROC and AUC to present model discrimination. We applied DeLong test to compare the AUCs of two models with and without the four‐way interaction. Also, the concordance index (*C*‐index), an average accuracy of predictive survival across follow‐up years, which ranges from 0.5 to 1.0, was calculated to estimate the predictive performance.

#### 2.4.5 Statistical analysis

Continuous variables were expressed as mean ± standard deviation (SD), and categorical variables were expressed in frequency (*n*) and proportion (%). Multiple comparisons were adjusted using the false discovery rate (FDR) method, as measured by the FDR‐*q* value (false discovery rate‐adjusted *q* value) [34], to control the overall false‐positive rate at 5% level. Statistical analysis was performed using R version 4.3.1 (The R Foundation of Statistical Computing, https://www.r‐project.org/).

## 3 Results

### 3.1 Sample characteristics of the study population

After quality control, our epigenome‐wide DNA methylation data were composed of 311 891 CpG probes from 992 early‐stage NSCLC patients; 524 patients (*N*_LUAD_ = 425 and *N*_LUSC_ = 99) were in the discovery phase and 468 patients (*N*_LUAD_ = 227 and *N*_LUSC_ = 241) were in the validation phase. A total of 609 patients had gene expression data. Demographic and clinical information for these patients is detailed in Table 1 and Table S1.

**Table 1:** Demographic and clinical descriptions for early‐stage NSCLC patients with DNA methylation data in five international study centers. 95% CI, 95% confidence interval; LUAD, lung adenocarcinoma; LUSC, lung squamous cell carcinoma; TCGA, The Cancer Genome Atlas.

Variable	Discovery phase					Validation phase	Combined dataset
	USA‐Harvard (*N* = 149)	Spain^a^ (*N* = 207)	Norway (*N* = 132)	Sweden (*N* = 36)	All (*N* = 524)	TCGA (*N* = 468)	Overall (*N* = 992)
Age (years)	67.78 ± 9.92	65.77 ± 10.66	65.4 ± 9.28	72.25 ± 7.22	66.7 ± 10.04	66.52 ± 9.3	66.61 ± 9.69
Pack‐year of smoking	51.49 ± 40.62	43.24 ± 31.72	26.93 ± 17.62	22.64 ± 25.19	40.06 ± 33.00	47.05 ± 28.40	43.36 ± 31.10
Sex							
Female	66 (44.30%)	99 (47.83%)	70 (53.03%)	22 (61.11%)	257 (49.05%)	185 (39.53%)	442 (44.56%)
Male	83 (55.70%)	108 (52.17%)	62 (46.97%)	14 (38.89%)	267 (50.95%)	283 (60.47%)	550 (55.44%)
Smoking status							
Never	18 (12.08%)	28 (13.53%)	16 (12.12%)	18 (50.00%)	80 (15.27%)	0 (0.00%)	80 (8.06%)
Ever	81 (54.36%)	113 (54.59%)	74 (56.06%)	11 (30.56%)	279 (53.24%)	323 (69.02%)	602 (60.69%)
Current	50 (33.56%)	66 (31.88%)	42 (31.82%)	7 (19.44%)	165 (31.49%)	145 (30.98%)	310 (31.25%)
Clinical stage							
I	102 (68.46%)	167 (80.68%)	92 (69.70%)	34 (94.44%)	395 (75.38%)	301 (64.32%)	696 (70.16%)
II	47 (31.54%)	40 (19.32%)	40 (30.30%)	2 (5.56%)	129 (24.62%)	167 (35.68%)	296 (29.84%)
Histology							
LUAD	96 (64.43%)	169 (81.64%)	132 (100.00%)	28 (77.78%)	425 (81.11%)	227 (48.50%)	652 (65.73%)
LUSC	53 (35.57%)	38 (18.36%)	0 (0.00%)	8 (22.22%)	99 (18.89%)	241 (51.50%)	340 (34.27%)
Chemotherapy							
No	140 (93.96%)	166 (91.21%)	101 (76.52%)	25 (89.29%)	432 (87.98%)	151 (75.88%)	583 (84.49%)
Yes	9 (6.04%)	16 (8.79%)	31 (23.48%)	3 (10.71%)	59 (12.02%)	48 (24.12%)	107 (15.51%)
Unknown	0	25	0	8	33	269	302
Radiotherapy							
No	130 (87.25%)	172 (94.51%)	131 (99.24%)	28 (100.00%)	461 (93.89%)	190 (95.48%)	651 (94.35%)
Yes	19 (12.75%)	10 (5.49%)	1 (0.76%)	0 (0.00%)	30 (6.11%)	9 (4.52%)	39 (5.65%)
Unknown	0	25	0	8	33	269	302
Adjuvant therapy^b^							
No	125 (83.89%)	157 (86.26%)	100 (75.76%)	25 (89.29%)	407 (82.89%)	146 (73.37%)	553 (80.14%)
Yes	24 (16.11%)	25 (13.74%)	32 (24.24%)	3 (10.71%)	84 (17.11%)	53 (26.63%)	137 (19.86%)
Unknown	0	25	0	8	33	269	302
Survival year^c^							
Median (95% CI)	6.61 (5.14–7.49)	3.83 (3.21–4.46)	5.40 (5.16–5.77)	3.25 (2.09–4.39)	5.01 (4.53–5.25)	0.58 (0.50–0.70)	2.31 (2.00–2.64)
Censoring rate	0.1879	0.5459	0.6894	0.5278	0.479	0.7607	0.6119

^a^ Spain center is a collaborative study center, containing samples from Spain, Italy, the UK, France, and the United States.

^b^ Adjuvant therapy included chemotherapy and/or radiotherapy.

^c^ Restricted mean survival time was given because median was not available; proportion of samples lost to follow‐up or alive at the end of study.

### 3.2 A significant four‐way interaction involving cg16658473_*SHISA9*_ identified in a two‐phase study

In the discovery phase, 39 pairs of significant four‐way interactions were identified (FDR‐*q* ≤ 0.05); whereas only one pair of interactions remained significant in the validation phase (*P* ≤ 0.05) and showed robust results in the combined data (Table S2). There was a statistically significant four‐way interaction effect of cg16658473_*SHISA9*_ with pack‐years of smoking, cg05293407_*TRIM27*_, and cg00060500_*KIAA0226*_ on NSCLC survival (discovery set: HR_interaction_ = 0.9993, 95% CI: 0.9990–0.9996, *P* = 3.08 × 10^−6^, FDR‐*q* = 0.027; validation set: HR_interaction_ = 0.9992, 95% CI: 0.9986–0.9998, *P* = 0.014; combined data: HR_interaction_ = 0.9995, 95% CI: 0.9993–0.9997, *P* = 3.06 × 10^−6^). However, the identified CpG probe (cg16658473) did not have significant marginal effects (Table S3). Annotation information for three CpG probes located in the genes *TRIM27*, *KIAA0226*, and *SHISA9* was presented in Table S4.

**Fig. 2:**
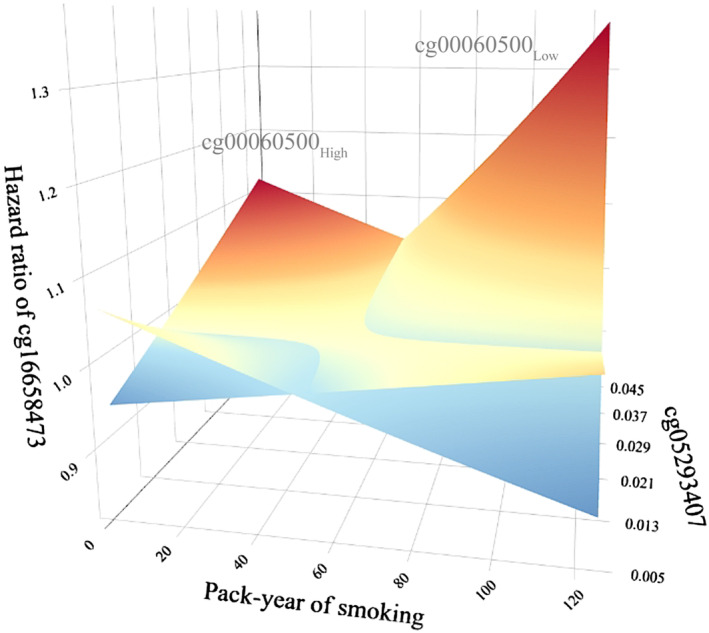
The 3D figure for visualization of four‐way interactions. Results of four‐way interaction among pack‐years of smoking and cg16658473_*SHISA9*_, cg05293407_*TRIM27*_, and cg00060500_*KIAA0226*_. The varying effects of cg16658473_*SHISA9*_ measured using HR at different smoking and cg05293407_*TRIM27*_ methylation levels in the subgroup with low and high level of cg00060500_*KIAA0226*_, respectively. HR, hazard ratio.

To visualize the four‐way interactions, we generated a 3D figure. Samples were divided into two subgroups based on the median level of cg00060500_*KIAA0226*_. Then, the effect of cg16658473_*SHISA9*_ on NSCLC survival measured by HR at different combinations of pack‐year of smoking and cg05293407_*TRIM27*_ methylation level was exhibited as a 3‐D surface in the subgroup with high and low expression of cg00060500_*KIAA0226*_, respectively (Fig. 2). In the low expression subgroup of cg00060500_*KIAA0226*_, as smoking intensity increases and methylation levels of cg05293407_*TRIM27*_ decrease, we observed an increased risk associated with cg16658473_*SHISA9*_. Conversely, in the high expression subgroup of cg00060500_*KIAA0226*_, as smoking intensity decreases and methylation levels of cg05293407_*TRIM27*_ increase, the effects of cg16658473_*SHISA9*_ are completely reversed.

### 3.3 Significant survival heterogeneity among TRIUNE subgroups

**Fig. 3:**
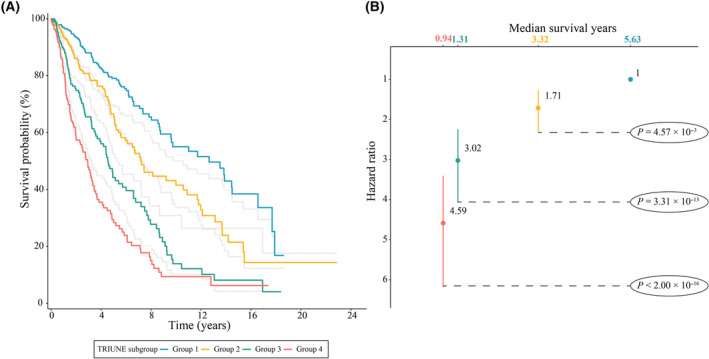
Comparison of overall survival time of patients among TRIUNE subgroups. (A) Kaplan–Meier survival curves for patients grouped by TRIUNE. Patients were categorized into four subgroups by using the quartiles of TRIUNE as the cutoffs. The number of patients in Group 1 to Group 4 was 248. The gray lines indicate the groups of patients based on the quartiles of the risk scores derived from a clinical model without the addition of the TRIUNE. (B) Hazard ratios and *P* values were derived from the Cox proportional hazards model for four groups of patients, where Group 1 was set to be reference group. Error bars indicate 95% confidence interval (CI).

**Fig. 4:**
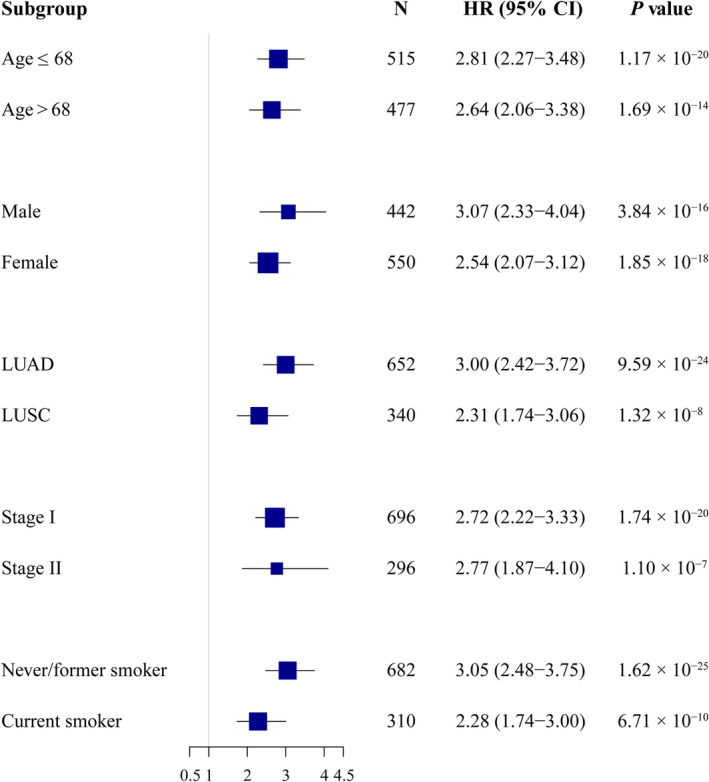
Forest plots of results from stratification analysis of TRIUNE. Hazard ratio (HR) with 95% confidence interval (CI) of TRIUNE on non‐small‐cell lung cancer (NSCLC) survival in different subgroups stratified by clinical characteristics. LUAD, lung adenocarcinoma; LUSC, lung squamous cell carcinoma. HR, 95% CI, and *P* value was derived from a Cox proportional hazards regression model. Error bars indicate 95% CI.

As depicted in Fig. 3, patients were stratified into four distinct subgroups according to the quartiles of TRIUNE, wherein Group 1 and Group 4 denoted as the subgroup with the smallest and largest risk, respectively. For median survival years, Group 1 was 5.63 years, followed by a decrease in each subsequent group, with the Group 4 having the shortest median survival (0.94, 95% CI: 0.75–1.18). Furthermore, in line with this trend, patients with higher TRIUNE exhibited a higher risk of mortality (HR_4 vs 1_ = 4.59, 95% CI: 3.40–6.18, *P* < 2.00 × 10^−16^; HR_3 vs 1_ = 3.02, 95% CI: 2.25–4.07, *P* = 3.31 × 10^−13^; HR_2 vs 1_ = 1.71, 95% CI: 1.27–2.31, *P* = 4.57 × 10^−4^). For comparison, we also divided patients into four subgroups based on the clinical score, a weighted linear combination of clinical variables, as shown in gray lines in Fig. 3. The risk stratification ability of TRIUNE was obviously superior to that of clinical scores, and the association between TRIUNE and overall survival remained significant in all subgroup analyses stratified by different clinical factors (Fig. 4), indicating it was a robustly significant score of lung cancer survival.

### 3.4 *Cis*‐regulatory genes were enriched in biological pathways

Genome‐wide correlation analyses identified 892 genes whose expression levels were significantly associated with the DNA methylation levels of these CpG probes involved in TRIUNE (FDR‐*q* ≤ 0.05). These genes were further found to be enriched in 20 KEGG pathways, which were primarily related to immune system processes (Fig. S2A). Moreover, GO enrichment analysis identified 83 significant biological process pathways, 8 cellular component pathways, and 12 molecular function pathways, many of which are related to immune system regulation (Fig. S2B–D).

### 3.5 A significant four‐way interaction involving *TRIM27*, *FYTTD1*, and *SHISA9* identified in genes *cis*‐regulated by CpG probes

We observed that *TRIM27*, *KIAA0226*, *FYTTD1*, and *SHISA9* were significantly *cis*‐regulated by CpG probes within 1 kb window (Table S5). For CpG probe cg00060500, it was mapped to both *TRIM27* and *KIAA0226*. Meanwhile, there was a significant correlation (*r* = 0.455, *P* = 5.21 × 10^−33^) between *KIAA0226* and *FYTTD1* (Fig. S3). We did not observe significant (*P* = 0.530) four‐way interaction among pack‐year of smoking, *TRIM27*, *KIAA0226*, and *SHISA9* (Table S6). Anyway, there is significant four‐way interaction among pack‐year of smoking, *TRIM27*, *FYTTD1*, and *SHISA9* (Table S7).

### 3.6 Four‐way interaction empowered prognostic prediction model

**Fig. 5:**
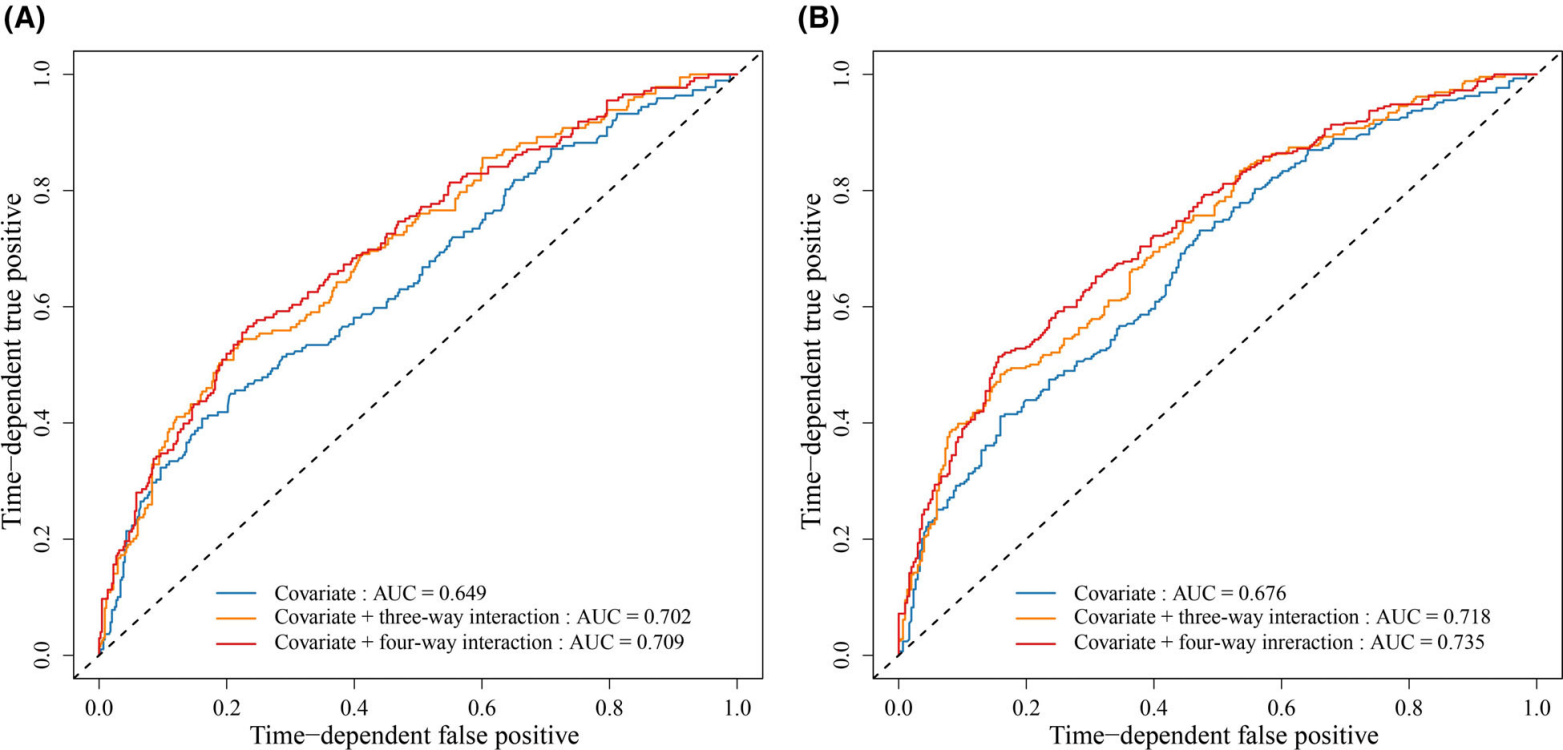
ROC curves were generated to compare the predictive performance of different models. Three models were: (a) one using only covariates (clinical variables); (b) one incorporating covariates and all elements of three‐way interactions (cg05293407_*TRIM27*_, pack‐year of smoking and another CpG probe) with FDR‐*q* ≤ 0.05; and (c) another one including covariates and a four‐way interaction (pack‐year of smoking, cg05293407_*TRIM27*_, cg00060500_*KIAA0226*_ and cg16658473_*SHISA9*_) with FDR‐*q* ≤ 0.05. (A) ROC for 3‐year survival prediction. (B) ROC for 5‐year survival prediction. AUC, area under the receiver operating characteristic curve; ROC, receiver operating characteristic. The dashed line represents the performance of a random classifier, corresponding to an AUC of 0.5. FDR‐*q*, false discovery rate‐adjusted *q* value.

As depicted in Fig. 5, initially, the predictive capacity of the model comprising solely demographic and clinical variables was found to be limited (AUC_3‐year_ = 0.649, 95% CI: 0.601–0.694; AUC_5‐year_ = 0.677, 95% CI: 0.633–0.719, *C*‐index = 0.625). Nonetheless, a slight but discernible improvement of AUC was observed with the inclusion of all elements of the three‐way interaction (pack‐year of smoking × cg05293407_*TRIM27*_ × cg00060500_*KIAA0226*_), with AUC_3‐year_ = 0.702 (95% CI: 0.656–0.745), AUC_5‐year_ = 0.718 (95% CI: 0.676–0.758), *C*‐index = 0.661. Moreover, the AUC was further improved through the incorporation of all elements of the four‐way interaction (pack‐year of smoking × cg05293407_*TRIM27*_ × cg00060500_*KIAA0226*_ × cg16658473_*SHISA9*_), with AUC_3‐year_ = 0.709 (95% CI: 0.663–0.753), AUC_5‐year_ = 0.735 (95% CI: 0.693–0.775), and *C*‐index = 0.673. The addition of the four‐way interaction term, compared to the clinical model, resulted in AUC increases of 9.23% (*P* = 1.74 × 10^−4^) and 8.52% (*P* = 2.00 × 10^−4^) for 3‐ and 5‐year survival, respectively.

## 4 Discussion

To our knowledge, we were among the first to conduct an epigenomic study on four‐way gene‐smoking interactions related to overall survival in early‐stage NSCLC patients. We identified one CpG probe (cg16658473_*SHISA9*_), by interacting with cg05293407_*TRIM27*_, cg00060500_*KIAA0226*_ and pack‐year of smoking, displaying a significant four‐way interaction effect on the overall survival of NSCLC. Moreover, the TRIUNE we developed revealed substantial survival heterogeneity among subgroups. We also identified a four‐way gene‐smoking interaction in gene expression level using genes *cis*‐regulated by CpG probes. Finally, the four‐way interaction moderately, but significantly, improved the discrimination ability of the prognostic prediction model.

Statistical G × G and G × E interactions, particularly at the epigenetic level, are crucial for understanding the molecular mechanisms underlying cancer progression [15, 16, 21, 22, 23, 33, 41, 42, 43, 44, 45, 46]. Taking the two‐way interaction as an example, the statistical interaction is the deviation between the joint effect of two factors and sum of their main effects, indicating a synergistic or antagonistic effect. On the other way, it can be also defined as the variation of the marginal effect of one factor across strata of another factor [41]. In this study, we focused on investigating these interactions through a rigorous two‐phase study and identified one four‐way G × E interaction. It is a common phenomenon that interaction effect among several factors is usually weaker than their own main effects [22, 23, 33]. Although two‐, three‐, and four‐way interaction effects are small, but they remain statistically significant, underscoring their robustness and relevance [22, 23]. Moreover, the 3D figure we generated visualized the differential effects of cg16658473_*SHISA9*_ at different levels of these variables. These findings underscore the presence of a complex four‐way interaction, which may offer valuable insights into the epigenetic regulation of lung cancer prognosis. Building on our previous work with two‐ and three‐way interactions, we found that including the four‐way interaction moderately enhanced the prediction accuracy for both 3‐ and 5‐year survival. However, the underlying biological mechanisms of four‐way interactions warrant further investigation.

In current study, we identified one novel gene, *SHISA9* (Shisa Family Member 9), contributing to the four‐way interaction. It was also called *CKAMP44*, acting as a protein coding gene whose coding proteins belongs to the Shisa protein family [47] and constitute the Cystine‐knot AMPA receptor‐modulating proteins (*CKAMPs*) family [48]. Emerging evidence indicates that *SHISA9* is intricately involved in immune modulation, particularly through its interactions with autophagy processes and the inhibitor of nuclear factor κB kinase subunit epsilon (IKKε) signaling pathway [49]. NF‐κB signaling pathway, including IKKε pathway, supports tumor survival by regulating apoptosis, promoting the formation of an inflammatory microenvironment, and affect survival outcomes in NSCLC [50, 51]. In 2021, Ke et al. [52] discovered that *SHISA9* was an independent risk factor for poor prognosis in LUSC patients. Therefore, *SHISA9* expression may modulate the IKKi signaling pathway, ultimately impacting NSCLC patients.

*TRIM27* played a crucial role in enhancing autophagy flux by activating transcription factor e‐box binding (TFEB), thereby contributing to the host's immune defense against intracellular pathogens [53]. Our previous study found that *KIAA0226* may interact with cigarette smoke, regulating the transcription of *Beclin‐1* and affecting the cross‐regulation between apoptosis and autophagy [23]. Thus, it is plausible that *SHISA9* interacts with *TRIM27*, *KIAA0226* and smoking, influencing the autophagy process and ultimately affecting lung cancer prognosis [54, 55]. Despite these findings, biological mechanisms underlying the four‐way interaction need to be more systematically studied.

Since DNA methylation may directly affect gene expression and impact cancer prognosis [56, 57], we further test the four‐way interaction at the gene expression level using genes *cis*‐regulated by CpG probes. Anyway, we merely observed interaction among *TRIM27*, *FYTTD1*, *SHISA9*, and pack‐year of smoking. *FYTTD1* (Forty‐two‐three Domain Containing 1), also called *UIF*, was found to play a crucial role on mRNA export [58, 59]. Given the interplay between mRNA export and autophagy [60], the interaction of *TRIM27*, *FYTTD1*, *SHISA9* and pack‐years of smoking may influence autophagy and, in turn, affect lung cancer prognosis [61]. Interestingly, while cg00060500 was associated with both *KIAA0226* and *FYTTD1*, only *FYTTD1* showed a significant interaction. Despite the significant correlation between *FYTTD1* and *KIAA0226*, their independent contributions to the interaction model likely differ due to distinct biological functions and the complexity of four‐way interactions. *FYTTD1* likely influenced mRNA transcription through its role in mRNA export, directly affecting gene expression and interacting more significantly with the other three factors to impact NSCLC survival. In contrast, while *KIAA0226* may interact with smoking to influence autophagy, the complexity of autophagy likely modulates or dilutes its effect, potentially explaining the lack of significance in the four‐way interaction. This difference in biological functions and interaction patterns may explain why *FYTTD1* showed a significant four‐way interaction, while *KIAA0226* did not. These findings underscore the variability in gene‐specific regulatory effects, highlighting the need for further investigation in NSCLC.

Notably, alongside the heterogeneous survival among the four subgroups, genes significantly associated with CpG probes were enriched in immune‐related pathways, including NF‐κB, T cell, and B cell signaling pathways, which may affect overall survival in NSCLC [62, 63, 64]. Moreover, enriched terms such as cytokine receptor activity and MHC protein binding, were reported to play essential roles in modulating immune responses, which could impact lung cancer prognosis [65, 66, 67, 68]. Our subgroup analysis further indicates that immune‐related pathways and biological functions may play pivotal roles in the progression of lung cancer.

There are some strengths in our study. First, we were among the first to conduct an epigenomic study on four‐way gene‐smoking interactions related to overall survival in early‐stage NSCLC patients. Next, the false positives were undergone critically appraised in our two‐phase study. During the discovery phase, the significant threshold was defined as FDR‐*q* ≤ 0.05 and meanwhile as *P* ≤ 0.05 in the validation phase. Therefore, the final false‐positive rate is already around 0.0025 (0.05 × 0.05). Then, we generated the 3‐D plot and forest plot to facilitate the understanding and interpretation of four‐way interaction. Moreover, incorporating four‐way interaction, we construct a prognostic model, which may aid in clinical decision‐making. Finally, in addition to identifying interactions between CpG probes, we also investigated the interactions between the genes *cis*‐regulated by these CpG probes.

We also have some limitations. First, we have only made a stratified analysis of TRIUNE and have not elucidated the biological mechanisms. Next, the TCGA cohort has a high censoring rate (76.07%) of survival time and thus the validation phase based on samples from TCGA population had low statistical power. Anyway, these signals confirmed significant in TCGA indicates what we observed is conservative and robust. Ultimately, as the majority of population included in this study was Caucasian (89%), the extrapolation of conclusions requires caution.

## 5 Conclusion

We identified a significant CpG probe (cg16658473_*SHISA9*_), together with cg05293407_*TRIM27*_, cg00060500_*KIAA0226*_ and pack‐year of smoking, to constitute a four‐way gene‐smoking interaction associated with NSCLC survival.

## Conflict of interest

The authors declare no conflict of interest.

## Author contributions

LC, DCC, RZ, and FC contributed to conception and design. LL, DCC, RZ, and FC contributed to financial support. LS, MMB, AK, MP, JS, ÅH, ME, DCC, and RZ contributed to data collection and quality control. LC, XW, NX, ZZ, XX, MX, YY, LL, DCC, and RZ contributed to data analysis and interpretation. LC and RZ contributed to manuscript writing. LC, XW, NX, ZZ, XX, MX, YY, LL, DCC, and RZ contributed to critical revision. All authors contributed to final approval of manuscript and accountable for all aspects of the work.

### Peer review

The peer review history for this article is available at https://www.webofscience.com/api/gateway/wos/peer‐review/10.1002/1878‐0261.13766.

## Supporting information


**Fig. S1.** Quality control processes for DNA methylation data.
**Fig. S2.** The functional enrichment analyses of genes significantly associated with CpG probes involving in TRIUNE.
**Fig. S3.** Correlation between *KIAA0226* and *FYTTD1*.
**Table S1.** Demographic and clinical descriptions of early‐stage NSCLC patients with gene expression data in four international study centers.
**Table S2.** Association results of four‐way interactions in the discovery phase, the validation phase, and the combined data.
**Table S3.** The association results of cg16658473_*SHISA9*_ derived from Cox proportional hazards model adjusted for covariates in NSCLC samples.
**Table S4.** Annotation information for three CpG probes located in the genes *TRIM27*, *KIAA0226*, and *SHISA9*.
**Table S5.** Correlation results between DNA methylation probes and corresponding gene expressions.
**Table S6.** Association results of the four‐way interaction among pack‐year of smoking, *TRIM27*, *KIAA0226*, and *SHISA9*.
**Table S7.** Association results of the four‐way interaction among pack‐year of smoking, *TRIM27*, *FYTTD1*, and *SHISA9*.

## Data Availability

The DNA methylation image data from the USA‐Harvard cohort can be requested from DCC. Data from the Spain, Norway, and Sweden cohorts can be requested from ME, ÅH, and JS, respectively. Alternatively, it can be retrieved from gene expression omnibus database (GSE39279, GSE66836 and GSE56044). TCGA: https://tcga‐data.nci.nih.gov; now hosted at GDC: https://portal.gdc.cancer.gov. GEO: https://www.ncbi.nlm.nih.gov/gds/.
