# Supplementary material for: A two‐phase epigenome‐wide four‐way gene–smoking interaction study of overall survival for early‐stage non‐small cell lung cancer
Source: Mol Oncol. 2024 Dec 4;19(1):173–87. doi: 10.1002/1878-0261.13766 (PMC11705728; doi:10.1002/1878-0261.13766)
Supplement: Supplementary file 1 — Fig. S1. Quality control processes for DNA methylation data. Fig. S2. The functional enrichment analyses of genes significantly associated with CpG probes involving in TRIUNE. Fig. S3. Correlation between KIAA0226 and FYTTD1. Table S1. Demographic and clinical descriptions of early‐stage NSCLC patients with gene expression data in four international study centers. Table S2. Association results of four‐way interactions in the discovery phase, the validation phase, and the combined data. Table S3. The association results of cg16658473 SHISA9 derived from Cox proportional hazards model adjusted for covariates in NSCLC samples. Table S4. Annotation information for three CpG probes located in the genes TRIM27, KIAA0226, and SHISA9. Table S5. Correlation results between DNA methylation probes and corresponding gene expressions. Table S6. Association results of the four‐way interaction among pack‐year of smoking, TRIM27, KIAA0226, and SHISA9. Table S7. Association results of the four‐way interaction among pack‐year of smoking, TRIM27, FYTTD1, and SHISA9. [file MOL2-19-173-s001.doc]

# Supplementary files

**Table S1.** Demographic and clinical descriptions of early-stage NSCLC patients with gene expression data in four international study centers.

| Characteristic | Harvard  (*N* = 26) | Norway  (*N* = 93) | Sweden  (*N* = 34) | TCGA  (*N* = 456) |
| --- | --- | --- | --- | --- |
| Age (years), mean ± SD | 66.39±9.45 | 65.94±9.05 | 71.85±7.24 | 66.58±9.14 |
| Sex, *n* (%) |  |  |  |  |
| Female | 11 (42.31) | 47 (50.54) | 21 (61.76) | 182 (39.91) |
| Male | 15 (57.69) | 46 (49.46) | 13 (38.24) | 274 (60.09) |
| Smoking status, *n* (%) |  |  |  |  |
| Never | 2 (7.69) | 11 (11.83) | 17 (50.00) | 0 (0.00) |
| Former | 15 (57.69) | 55 (59.14) | 11 (32.35) | 316 (69.3) |
| Current | 9 (34.62) | 27 (29.03) | 6 (17.65) | 140 (30.7) |
| TNM stage, *n* (%) |  |  |  |  |
| I | 20 (76.92) | 69 (74.19) | 32 (94.12) | 294 (64.47) |
| II | 6 (23.08) | 24 (25.81) | 2 (5.88) | 162 (35.53) |
| Histology, *n* (%) |  |  |  |  |
| LUAD | 17 (65.38) | 93 (100.00) | 26 (76.47) | 221 (48.46) |
| LUSC | 9 (34.62) | 0 (0.00) | 8 (23.53) | 235 (51.54) |
| Chemotherapy, *n* (%) |  |  |  |  |
| No | 26 (100.00) | 76 (81.72) | 23 (88.46) | 147 (76.17) |
| Yes | 0 (0.00) | 17 (18.28) | 3 (11.54) | 46 (23.83) |
| Unknown | 0 | 0 | 8 | 263 |
| Radiotherapy, *n* (%) |  |  |  |  |
| No | 22 (84.62) | 92 (98.92) | 26 (100.00) | 184 (95.34) |
| Yes | 4 (15.38) | 1 (1.08) | 0 (0.00) | 9 (4.66) |
| Unknown | 0 | 0 | 8 | 263 |
| Adjuvant therapy, *n* (%) |  |  |  |  |
| No | 22 (84.62) | 75 (80.65) | 23 (88.46) | 142 (73.58) |
| Yes | 4 (15.38) | 18 (19.35) | 3 (11.54) | 51 (26.42) |
| Unknown | 0 | 0 | 8 | 263 |
| Survival year |  |  |  |  |
| Median (95%CI) | 7.28 (5.41-9.27) | 5.27 (5.02-5.65) | 3.25 (2.08-4.39) | 0.58 (0.60-0.70) |
| Censored rate (%) | 26.92 | 67.74 | 50.00 | 76.32 |

NSCLC: non-small cell lung cancer; TGCA: The Cancer Genome Atlas; LUAD: lung adenocarcinoma; LUSC: lung squamous cell carcinoma; 95% CI: 95% confidence interval

**Table S2.** Association results of four-way interactions in the discovery phase, the validation phase, and the combined data.

| Variable | Discovery phase | | | | Validation phase | | | | Combined data | | | |
| --- | --- | --- | --- | --- | --- | --- | --- | --- | --- | --- | --- | --- |
|  | *beta* | 95% CI | | *P* value | *beta* | 95% CI | | *P* value | *beta* | 95% CI | | *P* value |
| cg05293407 | 10.4500 | 7.2240 | 13.6760 | 0.0012 | 20.7600 | 12.7000 | 28.8000 | 0.0096 | 8.3130 | 5.6640 | 10.9620 | 0.0017 |
| cg00060500 | 1.8010 | 1.1584 | 2.4436 | 0.0051 | 2.9100 | 1.5160 | 4.3040 | 0.0368 | 1.3810 | 0.8865 | 1.8755 | 0.0052 |
| cg16658473 | 0.4396 | 0.3201 | 0.5591 | 0.0002 | 0.7601 | 0.4878 | 1.0324 | 0.0053 | 0.3870 | 0.2895 | 0.4845 | 0.0001 |
| pack-year of smoking | 0.2937 | 0.1914 | 0.3960 | 0.0041 | 0.3072 | 0.1144 | 0.5000 | 0.1111 | 0.1722 | 0.0995 | 0.2449 | 0.0178 |
| cg05293407:cg00060500 | -1.2750 | -1.6612 | -0.8888 | 0.0010 | -2.0880 | -3.0033 | -1.1727 | 0.0225 | -0.9763 | -1.2828 | -0.6698 | 0.0015 |
| cg05293407:cg16658473 | -0.2955 | -0.3675 | -0.2235 | 4.01×10^-5^ | -0.5145 | -0.6984 | -0.3306 | 0.0052 | -0.2529 | -0.3132 | -0.1926 | 2.70×10^-5^ |
| cg00060500:cg16658473 | -0.0527 | -0.0670 | -0.0384 | 0.0002 | -0.0811 | -0.1128 | -0.0494 | 0.0106 | -0.0455 | -0.0567 | -0.0342 | 0.0001 |
| cg05293407:pack-year of smoking | -0.1935 | -0.2530 | -0.1340 | 0.0011 | -0.2432 | -0.3665 | -0.1199 | 0.0485 | -0.1223 | -0.1663 | -0.0783 | 0.0054 |
| cg00060500:pack-year of smoking | -0.0357 | -0.0482 | -0.0233 | 0.0042 | -0.0317 | -0.0525 | -0.0109 | 0.1273 | -0.0195 | -0.0276 | -0.0113 | 0.0174 |
| cg16658473:pack-year of smoking | -0.0095 | -0.0119 | -0.0072 | 4.08×10^-5^ | -0.0106 | -0.0152 | -0.0060 | 0.0211 | -0.0071 | -0.0088 | -0.0054 | 3.95×10^-5^ |
| cg05293407:cg00060500:cg16658473 | 0.0355 | 0.0271 | 0.0438 | 2.04×10^-6^ | 0.0541 | 0.0329 | 0.0754 | 0.0107 | 0.0297 | 0.0229 | 0.0365 | 1.24×10^-5^ |
| cg05293407:cg00060500:pack-year of smoking | 0.0239 | 0.0169 | 0.0309 | 0.0007 | 0.0246 | 0.0113 | 0.0379 | 0.0637 | 0.0142 | 0.0093 | 0.0190 | 0.0035 |
| cg05293407:cg16658473:pack-year of smoking | 0.0061 | 0.0048 | 0.0074 | 4.61×10^-6^ | 0.0076 | 0.0046 | 0.0106 | 0.0110 | 0.0046 | 0.0036 | 0.0057 | 6.16×10^-6^ |
| cg00060500:cg16658473:pack-year of smoking | 0.0011 | 0.0008 | 0.0014 | 0.0001 | 0.0011 | 0.0006 | 0.0016 | 0.0235 | 0.0008 | 0.0006 | 0.0010 | 3.39×10^-5^ |
| cg05293407:cg00060500:cg16658473:pack-year of smoking | -0.0007 | -0.0009 | -0.0006 | 3.08×10^-6^ | -0.0008 | -0.0011 | -0.0005 | 0.0144 | -0.0005 | -0.0006 | -0.0004 | 3.06×10^-6^ |

**Table S3.** The association results of cg16658473*_SHISA9_* derived from Cox proportional hazards model adjusted for covariates in NSCLC samples.

| Variable | HR | 95%CI | *P* |
| --- | --- | --- | --- |
| cg16658473*_SHISA9_* | 0.9999 | 0.9922~1.0001 | 0.9847 |

**Table S4.** Annotation information for three CpG probes located in the genes *TRIM27*, *KIAA0226*, and *SHISA9*.

| Probe | Gene | Location | Region | CpG island | Relation to CpG islands |
| --- | --- | --- | --- | --- | --- |
| cg05293407 | *TRIM27* | Chr6:28891967 | TSS200 | chr6:28890951-28892013 | Island |
| cg00060500 | *KIAA0226* | Chr3:197476529 | 5' UTR;  1^st^ Exon | chr3:197476262-197477556 | Island |
| cg16658473 | *SHISA9* | chr16:12998870 | Body | chr16:12994574-12997690 | Shore |

**Table S5.** Correlation results between DNA methylation probe and its corresponding gene.

| Index | CpG probe | Gene | *r* | *P* value |
| --- | --- | --- | --- | --- |
| 1 | cg05293407 | *TRIM27* | 0.1252 | 0.0018 |
| 2 | cg00060500 | *KIAA0226* | 0.0773 | 0.045 |
| 2 | cg00060500 | *FYTTD1* | 0.1827 | 4.70 × 10^-6^ |
| 3 | cg16658473 | *SHISA9* | 0.4022 | 1.68 × 10^-25^ |

**Table S6.** Association results of the four-way interaction among pack-year of smoking, *TRIM27*, *KIAA0226* and *SHISA9*.

| Variable | *beta* | 95%CI | | *P* value |
| --- | --- | --- | --- | --- |
| *TRIM27* | -0.0172 | -0.1892 | 0.1547 | 0.9202 |
| *KIAA0226* | 0.3645 | 0.1691 | 0.5599 | 0.0622 |
| *SHISA9* | -0.0719 | -0.3015 | 0.1577 | 0.7541 |
| pack_year | -0.0074 | -0.0115 | -0.0032 | 0.0768 |
| *TRIM27*:*KIAA0226* | 0.0909 | -0.1397 | 0.3215 | 0.6935 |
| *TRIM27*:*SHISA9* | 0.4548 | 0.2256 | 0.6840 | 0.0472 |
| *KIAA0226*:*SHISA9* | 0.1283 | -0.1244 | 0.3811 | 0.6116 |
| *TRIM27*:pack_year | -0.0013 | -0.0044 | 0.0018 | 0.6692 |
| *KIAA0226*:pack_year | -0.0045 | -0.0084 | -0.0006 | 0.2460 |
| *SHISA9*:pack_year | -0.0035 | -0.0087 | 0.0018 | 0.5060 |
| *TRIM27*:*KIAA0226*:*SHISA9* | 0.0701 | -0.1782 | 0.3184 | 0.7776 |
| *TRIM27*:*KIAA0226*:pack_year | 0.0026 | -0.0020 | 0.0072 | 0.5712 |
| *TRIM27*:*SHISA9*:pack_year | -0.0089 | -0.0131 | -0.0048 | 0.0320 |
| *KIAA0226*:*SHISA9*:pack_year | 0.0061 | 0.0010 | 0.0112 | 0.2326 |
| *TRIM27*:*KIAA0226*:*SHISA9*:pack_year | 0.0030 | -0.0018 | 0.0077 | 0.5303 |

**Table S7.** Association results of the four-way interaction among pack-year of smoking, *TRIM27*, *FYTTD1* and *SHISA9*.

| Variable | *beta* | 95%CI | | *P* value |
| --- | --- | --- | --- | --- |
| *TRIM27* | 0.1203 | -0.0707 | 0.3112 | 0.5289 |
| *FYTTD1* | 0.1861 | 0.0042 | 0.3679 | 0.3062 |
| *SHISA9* | -0.1079 | -0.3637 | 0.1479 | 0.6731 |
| pack_year | -0.0087 | -0.0128 | -0.0046 | 0.0352 |
| *TRIM27*:*FYTTD1* | -0.0446 | -0.2338 | 0.1446 | 0.8135 |
| *TRIM27*:*SHISA9* | 0.7034 | 0.4496 | 0.9571 | 0.0056 |
| *FYTTD1*:*SHISA9* | 0.0918 | -0.0990 | 0.2826 | 0.6306 |
| *TRIM27*:pack_year | -0.0061 | -0.0100 | -0.0021 | 0.1249 |
| *FYTTD1*:pack_year | 0.0007 | -0.0025 | 0.0040 | 0.8152 |
| *SHISA9*:pack_year | -0.0048 | -0.0104 | 0.0007 | 0.3860 |
| *TRIM27*:*FYTTD1*:*SHISA9* | -0.2143 | -0.4798 | 0.0512 | 0.4195 |
| *TRIM27*:*FYTTD1*:pack_year | 0.0053 | 0.0018 | 0.0089 | 0.1328 |
| *TRIM27*:*SHISA9*:pack_year | -0.0175 | -0.0228 | -0.0122 | 0.0010 |
| *FYTTD1*:*SHISA9*:pack_year | 0.0040 | -0.0002 | 0.0082 | 0.3371 |
| *TRIM27*:*FYTTD1*:*SHISA9*:pack_year | 0.0119 | 0.0065 | 0.0173 | 0.0285 |

**Fig. S1.** Quality control processes for DNA methylation data. Quality control procedures were applied to Harvard, Spain, Norway, Sweden, and The Cancer Genome Atlas (TCGA) samples using the same pipeline.


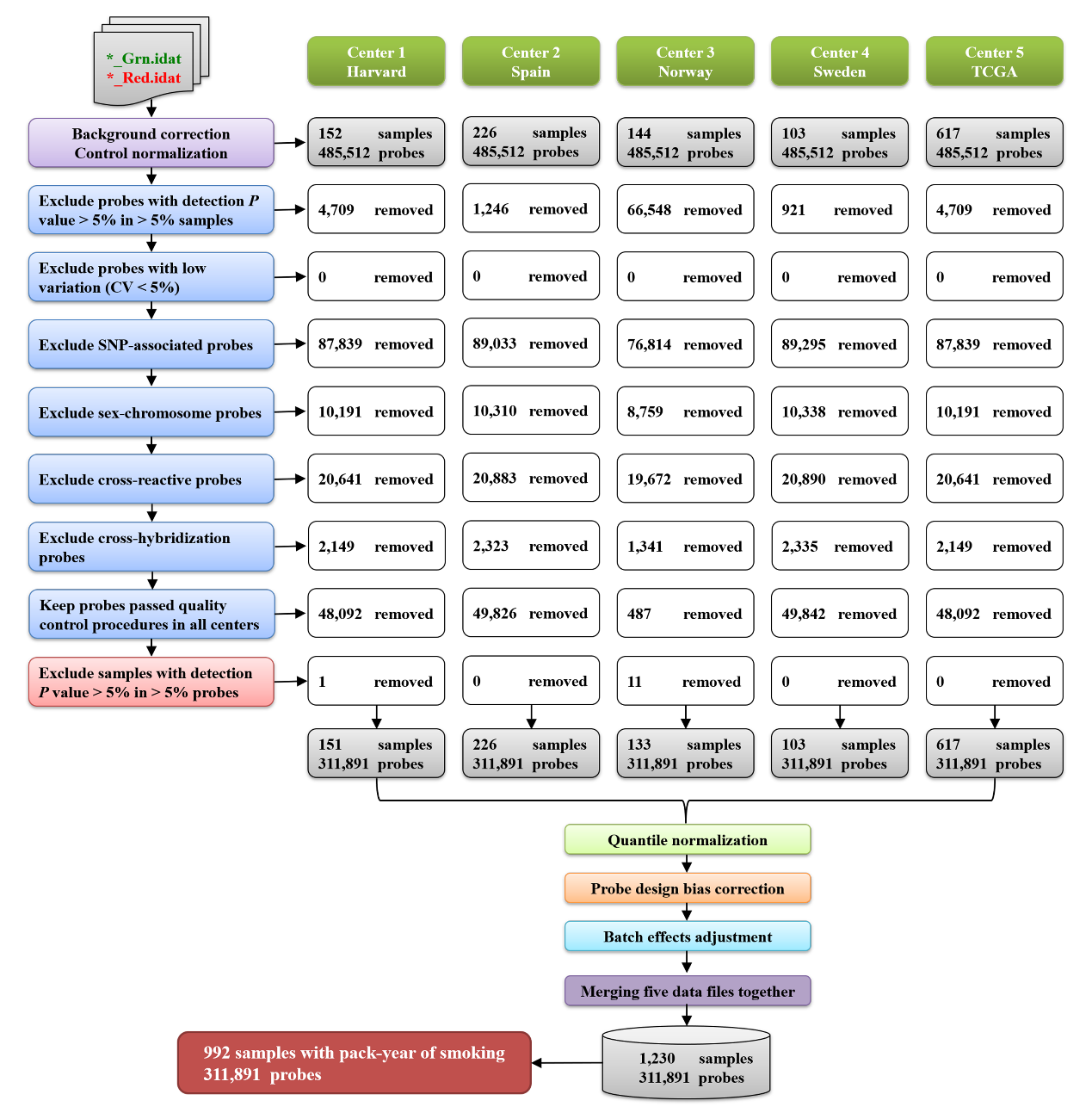


**Fig. S2.** The functional enrichment analyses of genes significantly associated with CpG probes involving in TRIUNE.


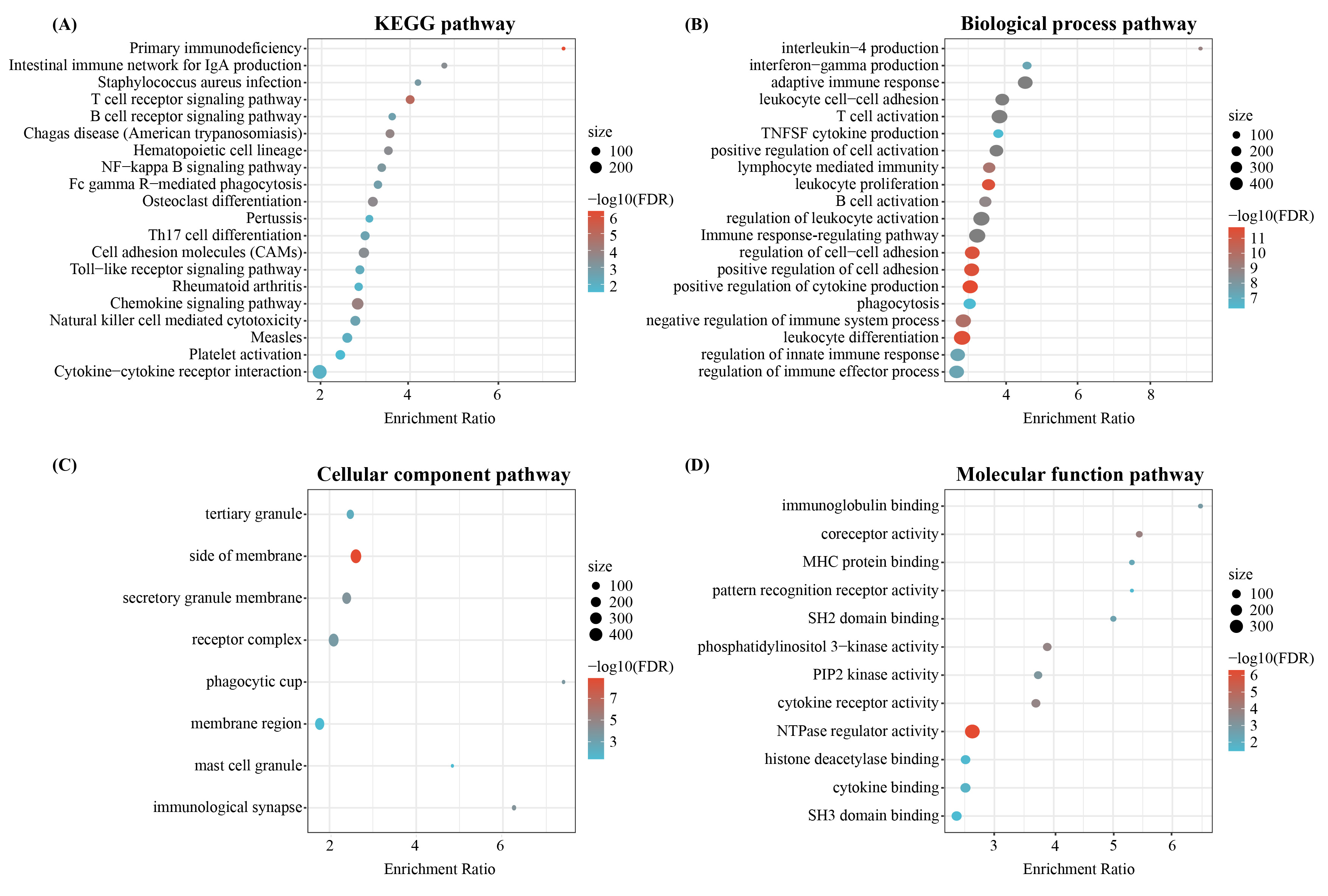


**(A)** Top 20 significant KEGG pathways. **(B)** Top 20 significant biological process pathways. **(C)** Top 8 significant cellular component pathways and **(D)** top 12 significant molecular function pathways.

**Fig. S3.** Correlation between *KIAA0226* and *FYTTD1*.


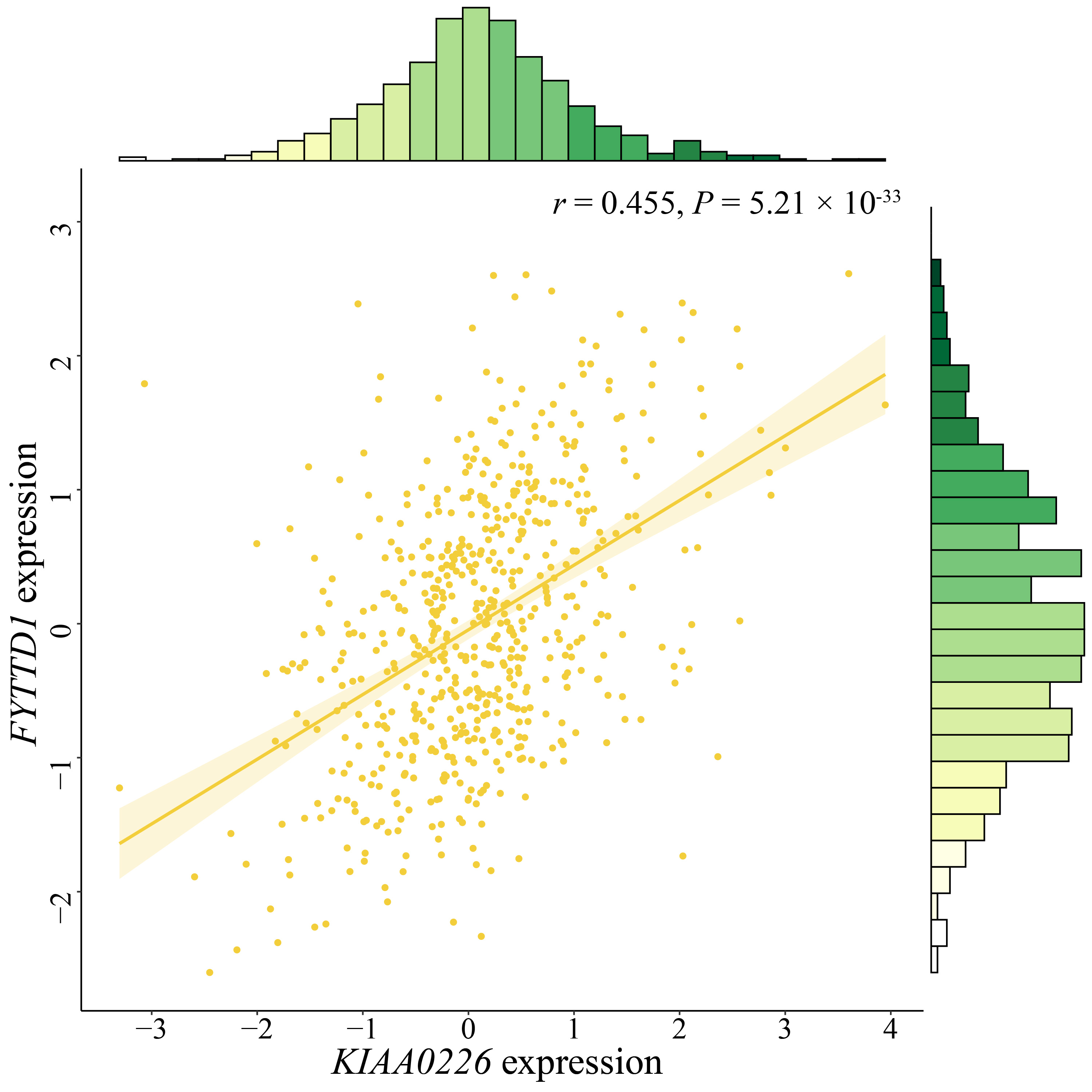


Correlation coefficient and *P* value were derived from Pearson correlation analysis. Histograms on top and sides were distributions of the two gene expressions.
